# Efficacy of a palliative care program in critically ill patients

**DOI:** 10.1186/cc10202

**Published:** 2011-06-22

**Authors:** SCF Ribeiro, ASRG Mazutti, SC Furlan, IP Campos, JF Seixas, APS Tavares, DT Noritomi

**Affiliations:** 1 Hospital Paulistano, São Paulo - SP, Brazil

## Introduction

For some critically ill patients, ICU treatment is more burdensome than beneficial and is inconsistent with these people's values, goals and preferences. Integration of palliative care in the ICU can help address this issue. We developed in our hospital a proactive palliative care program focusing on critically ill patients with chronic limitations aiming at: timely implementation of care plans that are realistic, appropriate and consistent with the patients' preferences and reductions in use of nonbeneficial treatments, thus also reducing the lengths of stay in ICU.

## Objective

To describe onset of a new palliative care program in a private hospital ICU.

## Methods

All critically ill patients with Karnofsky score <40% were evaluated by the multidisciplinary team when ICU transfer was considered or, when this was not possible, soon after arriving in the ICU. In the first familiar conference, the main family surrogate was identified and the patient's or family surrogate's preferences concerning advanced life support and end of life were discussed according to the clinical situation. Revaluation of these decisions was performed whenever necessary, according to the multidisciplinary team or family surrogate. Specific forms were filled to ensure adequate communication with the remaining hospital staff and for data acquisition.

## Results

Between November 2010 and January 2011, 61 patients were included in our palliative care program. The patients' median age was 78 (range: 38 to 101) years, with a slight predominance of women (54%). The main reason for palliative care was severe dementia. All patients had severe cognitive impairment, so all decisions were discussed solely with family surrogates. The program was started at the ICU in 52 (85%) and at the ER in nine (15%) cases. In the nine cases started outside the ICU, an ICU admission was avoided in eight (89%) of them. In 79% of the cases, decisions were made to withhold or withdraw some kind of life support. There were three family-assisted withdrawals of mechanical ventilation in the ICU. Several family surrogates reported that their decision was based on previously expressed patient's wishes.

## Conclusion

A proactive palliative care program focusing on critically ill patients with chronic and irreversible limitations is feasible and results in a better alignment of a patient's or their family's wishes and medical-related decisions and attitudes.

